# Direct Air CO_2_ Capture via Hot Water Cycling of Poly(ethyleneimine)/Silica Hollow Fiber Sorbents

**DOI:** 10.1021/acs.iecr.6c02807

**Published:** 2026-09-16

**Authors:** Zachary S. Campbell, Steven Schlosser, João Marreiros, William J. Koros, Christopher W. Jones, Matthew Realff, Ryan P. Lively

**Affiliations:** School of Chemical and Biomolecular Engineering, 1372 Georgia Institute of Technology, 311 Ferst Dr, Atlanta, Georgia 30332, United States

## Abstract

Hollow fiber sorbents containing amine-infused mesoporous silicas are promising materials for CO_2_ capture from ambient air due to their ease of heat integration and low pressure drops. This advanced sorbent design permits a wide range of heat source integration options, though limited experimental insights are found in the literature. In this study, hot water is utilized as the heat source for regenerating poly­(sulfone)-silica-poly­(ethyleneimine) fibers prepared with a hybrid poly­(vinylidene chloride)/styrene-butadiene lumen barrier. Fiber sorbents with this novel polymer and lumen formulation are characterized and tested using breakthrough and desorption measurements to evaluate the optimal adsorption and desorption times. Rapid temperature swing adsorption cycles are completed to evaluate the stability, productivity, and swing capacities of the fiber sorbents. In the final cycle, a swing capacity of 0.72 mmol g_fiber_^‑1^ under 400 ppm CO_2_ flow at 22 °C and 45% relative humidity was reached, with a CO_2_ recovery of 68%.

## Graphical abstract

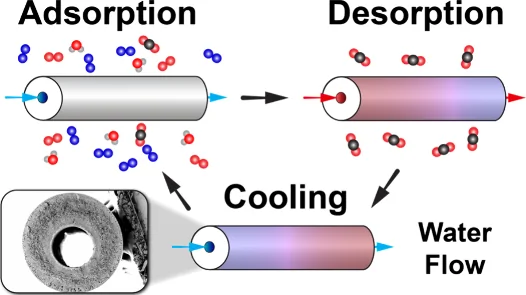

## Introduction

Solid adsorbents provide several notable benefits in terms of direct air capture (DAC) applications, including low regeneration temperatures compared to other technologies (e.g., aqueous scrubbers) and the potential for incorporation into structured contactors to minimize pressure drop.^1,2^ A wide range of solid sorbent materials has been explored, including zeolites, ion exchange resins, and amine-infused or functionalized porous materials (e.g., SiO_2_, metal oxides, MOFs, COFs).^3–11^ Of these materials, polyamine-infused amorphous silicas are especially interesting, as the components are inexpensive, readily available at a commercial scale, and are easily manufactured. Furthermore, they may be easily incorporated into structured contactors for efficient exposure to air. These hybrid polyamine-SiO_2_ materials have already been incorporated into monoliths,^12,13^ laminates,^14,15^ and fibers^16,17^ for CO_2_ capture applications, and all of these form factors are appealing due to their high volumetric loadings of sorbent and scalability. However, *hollow* fiber sorbents are especially intriguing due to the possibility of direct thermal contacting and heat integration by flowing a variety of thermal media, including water, steam, or another heat transfer fluid through the fiber bore, functionally combining an efficient adsorption system with a heat exchanger. Hollow fiber sorbents have already been utilized for effective heat integration in rapid temperature swing adsorption processes with both zeolite-loaded^18^ and poly­(ethyleneimine) (PEI)-infused silicas for post-combustion CO_2_ capture applications.^19^ However, poly­(sulfone)-based PEI/silica hollow fibers have not yet been evaluated for DAC rapid temperature swing adsorption (RTSA) applications with hot water integration, particularly using a low-grade heat source (e.g., 86 °C water) while evaluating the fibers in full adsorption-desorption-cooling cycles.

In this work, we present the efficient thermal cycling of poly­(sulfone) hollow fiber sorbents containing poly­(ethyleneimine)-infused amorphous silica using hot and cold water. The heating and cooling times of the fibers were measured and the breakthrough performance was evaluated. Adsorption and cooling cycles were utilized to generate productivity curves and determine potentially optimal cycling times under moderate relative humidity conditions. The prepared contactors were utilized to conduct 10 adsorption/desorption cycles using the estimated parameters and evaluate the short-term cyclic stability of the fibers.

## Materials and Experimental Methods

### Materials

Poly­(sulfone) (PSF) (Udel^®^ P-3500 NT11) was purchased from Syensqo. Silica (Syloid^®^ C803) was purchased from W. R. Grace & Co. Poly­(vinylidene chloride) latex (PVDC, L905B) was provided by Asahi Kasei Corp. The styrene-butadiene copolymer mixture (FlexSeal^®^ clear) was purchased from Home Depot. N-methyl pyrrolidone (NMP, 99%) was purchased from Oakwood Chemical. Poly­(vinylpyrrolidone) (PVP, 55 kDa MW), branched poly­(ethyleneimine) (PEI, 800 Da MW), poly­(ethylene glycol) (PEG, Mn ∼ 200), and poly­(propylene glycol) (PPG, Mn ∼ 725) were purchased from Sigma-Aldrich. Methanol (ACS) and hexane (ACS) were purchased from VWR. Nitrogen (99.999%), compressed air, and 400 ppm CO_2_ (balance N_2_) were purchased from Airgas. Epoxy (3M Scotch-Weld DP100) was purchased from McMaster-Carr. Deionized water was produced onsite using a Barnstead B-Pure system. All materials were used as received.

### Fiber Spinning

The PSF/silica spinning dope was prepared by combining dry silica, NMP, PSF, and PVP using the previously reported procedures.^16,17^ Fibers were prepared using a dry jet, wet quench spinning method^16,17^ using the dope composition and spinning parameters found in Table 1. Once the spin was complete, the fibers were washed in DI water for three days to remove residual NMP and PVP. The water used for the solvent exchange was replaced after each 24-hour period. The fibers were then solvent exchanged into methanol by conducting three 20-minute washes to remove any water, after which they were permitted to dry under ambient conditions overnight. The spinning process and setup are depicted in Figure 1.

**Figure 1:**
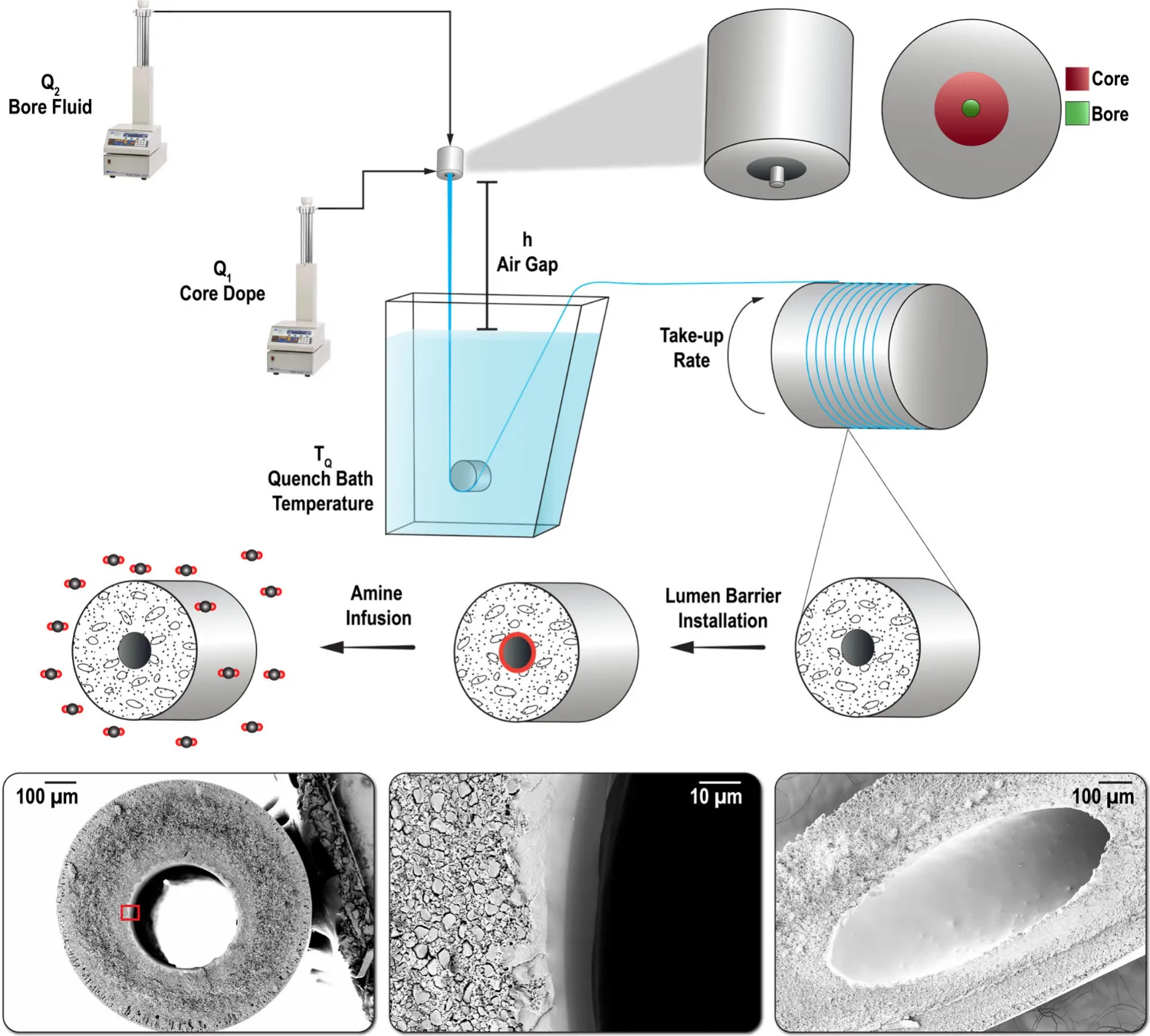
Diagram of the fiber spinning setup and preparation procedure. Left SEM image: cross-section of fiber with lumen barrier installed. Center SEM image: high magnification cross-section of lumen barrier. Right SEM image: diagonally cut fiber to expose lumen surface.

**Table 1:** Spinning Dope Composition and Parameters

Dope Component	wt %	Spin Parameter	
N-methyl-2-pyrrolidone	67.4	Dope flow rate	600 mL h^‑1^
		Bore fluid flow rate	100 mL h^‑1^
Syloid® C803	14.1	Bore fluid composition	50/50 PEG/PPG
		Spinneret temperature	23 °C
Polysulfone	11.5	Quench bath temperature	55 °C
		Air gap	3 cm
Poly(vinylpyrrolidone)	7.0	Take up rate	17.5 m min^‑1^

### Lumen Barrier Installation

To install the lumen barrier, the fibers were first installed in a shell-and-tube module (Supporting Information Figure S1). The fiber bores were pre-saturated using 1000 sccm humid N_2_ for 30 minutes, after which 30 mL of a 50/50 v/v% PVDC / DI water solution was fed through the bore of the fibers at a rate of 600 mL fiber^‑1^ h^‑1^ using a syringe pump (ISCO 500D). While the PVDC solution was fed through the module, the outlet end of the module was immersed in an n-hexane bath to prevent premature solidification of the PVDC mixture. Once the full 30 mL of solution was exhausted, 60 psi 50% relative humidity (RH) N_2_ was used to eject excess PVDC solution from the fiber bores and begin drying the nascent PVDC barrier coatings/films. The PVDC barriers were dried for 6 hours under humid N_2_ flow, followed by drying under ambient conditions for 15 hours. A second PVDC coat was then applied at the opposite end of the module using the same procedure. After drying the second coat, modules were annealed in a vacuum oven overnight at 90°C, following the more exhaustive description originally reported by Lively et al.^20^ After annealing, a secondary styrene-butadiene barrier coating was applied by flowing 10 mL of a 50 wt% styrene-butadiene/hexane solution through the fiber bores. Humid N_2_ was used to remove residual solution from the fiber bores, and the styrene-butadiene layer was allowed to dry and cure under ambient conditions overnight. Micrographs of the lumen barriers are shown in Figure 1 and Supporting Information Figures S2 and S3. The barriers were characterized by flowing hot water through the bores and measuring the water in the shell-side of the module using a 200 sccm N_2_ sweep. The water flux was found to be 0.00057 mol kg_fiber_^‑1^ s^‑1^, which was determined to be sufficiently low for use in subsequent measurements.

### Module Wash

The module was washed prior to PEI infusion due to the acidic nature of the PVDC solution (e.g. presence of acidic solutes and surfactants) which may be pulled into the fiber walls via capillary forces. Approximately 30 mL of water was flowed through the shell side of the module overnight using a peristaltic pump (Kamoer NKCP-S04B-US) to remove any contaminants. Following the water wash, 40 mL of methanol was flowed through the module shell side for approximately 30 minutes for residual water removal.

### Amine Infusion

The modules were amine-infused following the lumen barrier installation to avoid the degradation or leaching of the infused PEI during the installation procedure. A 15 wt% PEI solution in methanol was prepared by adding 4.5 g of PEI to a vial with 25.5 g of methanol. The solution was mixed until homogeneous, after which it was attached to the peristaltic pump and recirculated through the module shell side for 8 hours. It is important to note that the module was oscillated during the first two minutes to remove any trapped air bubbles that could impede liquid/solid contact and limit PEI diffusion into the fibers, after which the module was kept horizontal for the duration of the amine infusion. After the infusion was complete, the PEI/methanol solution was removed and the shell side of the module was washed repeatedly with hexane.

### Temperature Measurements

Temperatures were measured at the module inlet and outlet, as well as the fiber exterior in the center of the module to evaluate the heating and cooling rates as a function of water flow rate. Hot water (86 °C) was fed to the module at 30 mL min^‑1^ (10.2 cm s^‑1^) and the temperature response was measured until steady state was reached, after which the water feed was switched to cold water (22 °C) at the same flowrate. Repeat measurements were also conducted using 10 mL min^‑1^ (3.4 cm s^‑1^) and 20 mL min^‑1^ (6.8 cm s^‑1^) water flow rates.

### Breakthrough Measurements

Modules were activated by flowing 86°C water through the fiber bores at a rate of 30 mL min^‑1^ under a 200 sccm dry nitrogen sweep. After desorption, the hot water flow was switched to ambient (22 °C) water, and the module was allowed to cool under an inert N_2_ sweep. Once at 22 °C, a mixture of 500 sccm dry N_2_ and 500 sccm humid (90% RH) N_2_, resulting in a 45% RH feed stream, was introduced to the module to pre-saturate the fibers with water. The flow was then switched to a mixture of 500 sccm dry and 500 sccm humid (90% RH) 400 ppm CO_2_. The outlet CO_2_ concentration was measured using an infrared CO_2_ sensor (Li-COR Li-850) until pseudoequilibrium (i.e., C/C_0_ → 0.95) was reached. Breakthrough measurements were repeated in triplicate on a single module.

### CO_2_ Desorption Measurements

Following each breakthrough experiment, CO_2_ desorption was promoted by the same procedure as the pre-activation step while the shell side CO_2_ concentration was measured using a LiCOR Li-850 detector.

### Cyclic Measurements

Cyclic adsorption/desorption measurements were collected by functionally combining the procedures for the breakthrough and CO_2_ desorption measurements. No pre-saturation was used to more accurately simulate an actual temperature swing cycle. Each cycle was started by feeding N_2_ balanced 400 ppm CO_2_ (500 sccm dry, 500 sccm humid) to the module for 33 minutes. The feed gas was then switched to 1000 sccm dry N_2_ and hot water feed was introduced to the module to initiate the desorption process. After desorbing for 13 minutes, the water feed was switched from hot to cold and the module was permitted to cool for 2 minutes before switching to 400 ppm CO_2_ feed for the subsequent adsorption step. This procedure was repeated for a total of 10 cycles. The adsorption, desorption, and cooling times utilized for these cyclic measurements were carefully selected based on experimentally collected heating/cooling rates, hot water desorption kinetics, and adsorption productivities, which are fully discussed later in this manuscript.

### Characterization

Combustion thermogravimetric analysis (TGA) experiments were completed on bare PSF/C803 fibers, lumen-coated fibers, and PEI-infused lumen-coated fibers using a TA Instruments TGA Q500 to evaluate the sorbent loading. Samples were activated for 180 minutes under N_2_ flow at 100 °C, after which the feed gas was switched to compressed air and the temperature was ramped at 10 °C min^‑1^ to 600 °C with a 120-minute isothermal hold. N_2_ physisorption measurements were completed using a Micromeritics ASAP 2020. Samples were degassed at 110 °C under vacuum (<10^‑2^ kPa) for 12 hours before each measurement. Scanning electron microscope (SEM) images of the bare and lumen-coated fibers were collected using a Zeiss Ultra60 FE-SEM. Samples were cryo-fractured in liquid N_2_ to provide clean cross-sectional faces for imaging or were cut diagonally to present exposed lumen surfaces.

## Results and Discussion

Once modules were constructed and the lumen barrier was installed, initial experiments were completed to evaluate the heating and cooling rates of the module relative to the bore-side water flow rate. Figure 2 shows the heating and cooling profiles at the bore-side inlet and outlet, as well as the external temperature of the fiber bundle inside the module for flow rates of 10 (Figure 2A), 20 (Figure 2B), and 30 mL min^‑1^ (Figure 2C). The temperature profiles and final temperatures for the 20 and 30 mL min^‑1^ water flow rates were comparable, though the temperatures in the 30 mL min^‑1^ experiment approached steady state slightly faster with smaller thermal variance between the inlet and outlet water temperatures. In both cases, the modules reached thermal steady state/equilibrium in approximately 10 minutes, though the center fiber temperature continued to increase modestly afterwards. The relatively long time required for thermalization of the module, even at the higher flow rates, is attributable to several factors, specifically the relatively small fiber mass relative to the stainless-steel casing, significant heat loss from the casing to the environment despite its insulation, and the insulating fiber walls. The 10 mL min^‑1^ flow rate resulted in a noticeably longer equilibration time, requiring nearly 15 minutes to reach equilibrium, with lower maximum temperatures at each thermocouple location. This is explained by the longer residence time in the unheated segments of tubing relative to the higher flow rates.

**Figure 2:**
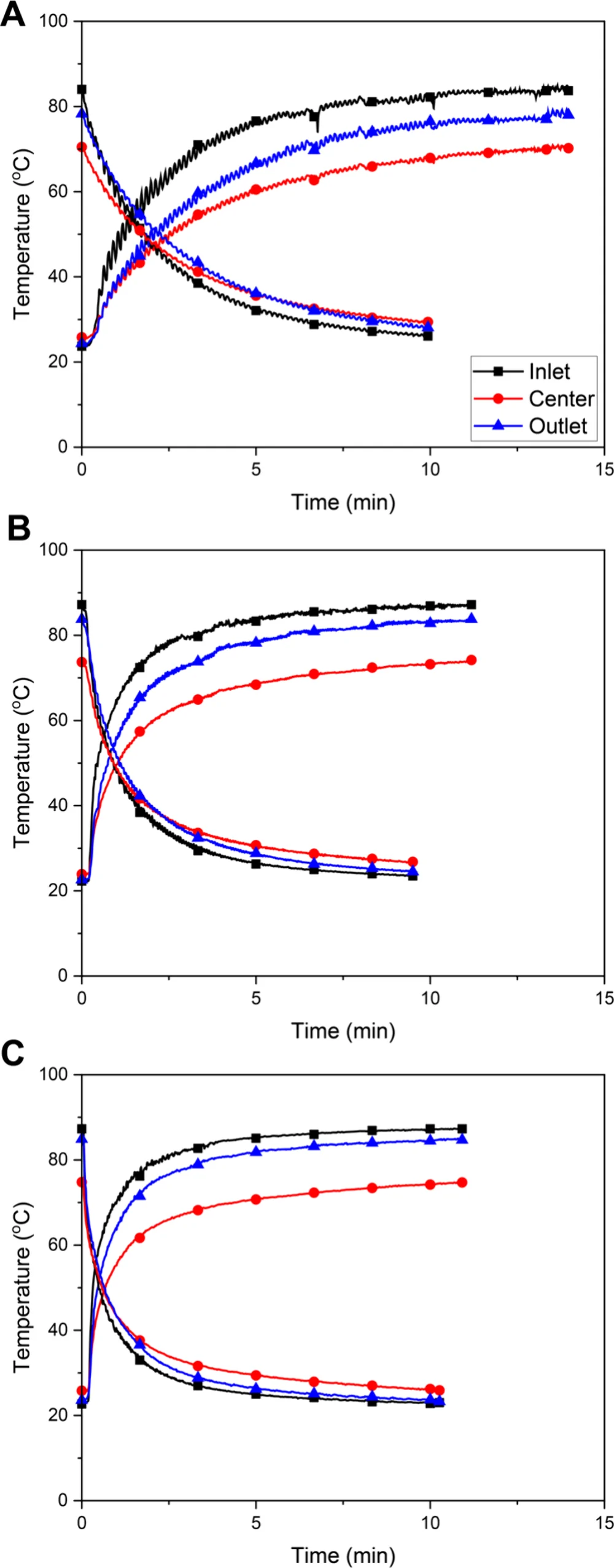
Heating and cooling curves at the inlet (black), outlet (blue), and center (red) for A) 10 mL min^‑1^, B) 20 mL min^‑1^, and C) 30 mL min^‑1^ water feed flow rates.

Notably, in all cases the center fiber temperature remained approximately 10 °C cooler than the inlet water temperature, which is likely attributable to the partial exposure of the center thermocouple to the air annulus on the shell-side of the module. It is also worthwhile to note that the relatively small quantity of fibers, especially relative to the mass of the stainless-steel module, may result in slower heating/cooling profiles and reduced temperatures than would be observed at larger scales.

The cooling curves exhibited smaller differences compared to the heating curves, due in part to the natural heat loss to the ambient environment, which functions as an infinite heat sink and results in greater consistency in cooling rates. The fiber temperature dropped below 40 °C in less than 5 minutes at each flow rate, with both 20 and 30 mL min^‑1^ dropping below 40 °C in less than 2.5 minutes. This rapid cooling is critically important, as it is vital to cool down amine-based sorbents prior to re-exposure to air to reduce oxidation rates and extend sorbent operating lifetime as much as possible.

After testing the heating and cooling curves, the fiber uptakes were evaluated using breakthrough measurements. A high flow rate (1000 sccm) was utilized to emulate the air velocities commonly targeted in pilot- and commercial-scale DAC facilities, usually in the range of 3-5 m s^‑1^, and provide reasonable estimates for fiber module productivity and adsorption times. A feed composition of 400 ppm CO_2_ balanced with nitrogen at 45% RH was selected as a proxy for atmospheric air conditions and used in all breakthrough testing trials. Figure 3 shows the recorded breakthrough curves and integrated CO_2_ uptakes for each of the replicate measurements. Feed (CO_2_) slip was observed immediately in each of the breakthroughs due to the high flow rates (0.85 m s^‑1^ superficial velocity). At this velocity, the volumetric turnover frequency was approximately 0.001 min^‑1^, meaning that the shell side gas volume was replaced 15 times per second. After the initial slippage, the measured CO_2_ concentration increased nearly linearly until approximately 60 minutes, where the slope began decreasing as the fibers approached saturation. Pseudoequilibrium (i.e., C/C_0_ → 0.95) was reached after approximately 100 minutes, with uptakes reaching 1.25 mmol g_fiber_^‑1^ (Figure 3B), with good agreement across all the measurements. Pressure drop represents an important consideration in the implementation of contactors for DAC, especially at higher flow rates. Measurements in similar parallel-flow tubular module configurations have been previously reported by Kong et al.^16^ and Sujan et al.^17^

**Figure 3:**
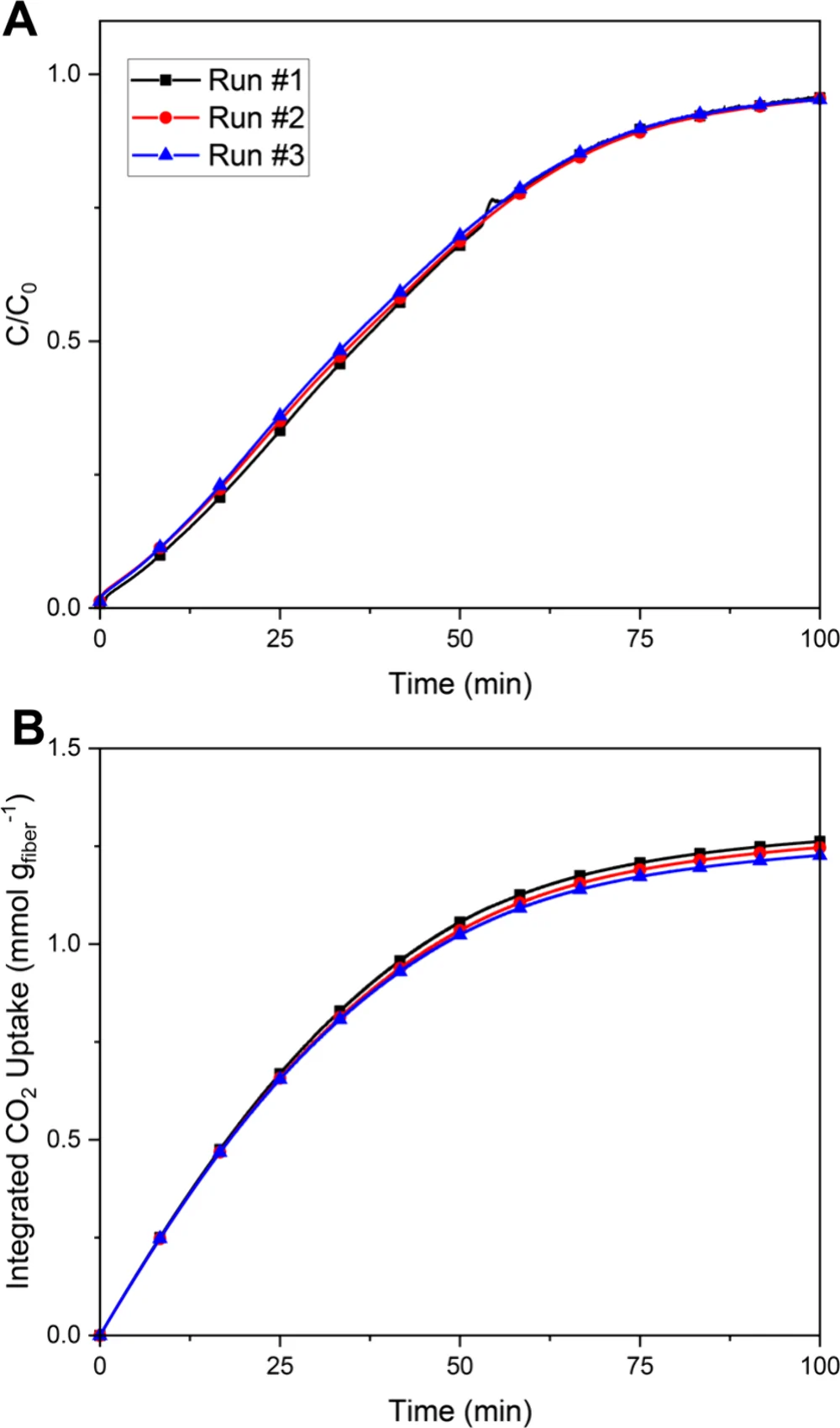
A) Breakthrough measurements and B) integrated CO_2_ uptake curves. Module length: 16.2 cm, gas flow rate: 1000 sccm, CO_2_ concentration: 400 ppm CO_2_, adsorption temperature: 22 °C

Following each breakthrough measurement, CO_2_ was desorbed by flowing 86 °C water through the fiber bores and removed with a 200 sccm dry N_2_ sweep until the CO_2_ outlet concentration dropped below 30 ppm. It should be noted that in this context, the inert sweep was utilized to facilitate measurement using the Li-COR Li-850 sensor. In an actual DAC deployment, alternative strategies (e.g., vacuum, pure CO_2_ purge) would be applied to ensure a CO_2_ stream of sufficient purity would be produced by the process. The shell-side CO_2_ concentration was measured and used to evaluate the rate of CO_2_ desorption (Figure 4A). Resulting CO_2_ concentration profiles revealed fast desorption response with spiking CO_2_ concentrations peaking at approximately 1.5 minutes after desorption was initiated and quickly decreasing thereafter. The bulk of the CO_2_ desorption was recorded within the first 10 minutes, notably slower than the steam desorption results reported by Kong et al. (∼3 minutes),^16^ likely due to the sensible heat of the large stainless-steel module shell coupled with the lower energy intensity of the warm water flow deployed in our tests. Figure 4B shows the integrated CO_2_ desorption curves for each run, with an average of 1.17 mmol CO_2_ g_fiber_^‑1^ desorbed, in reasonable agreement with the measured uptake values.

**Figure 4:**
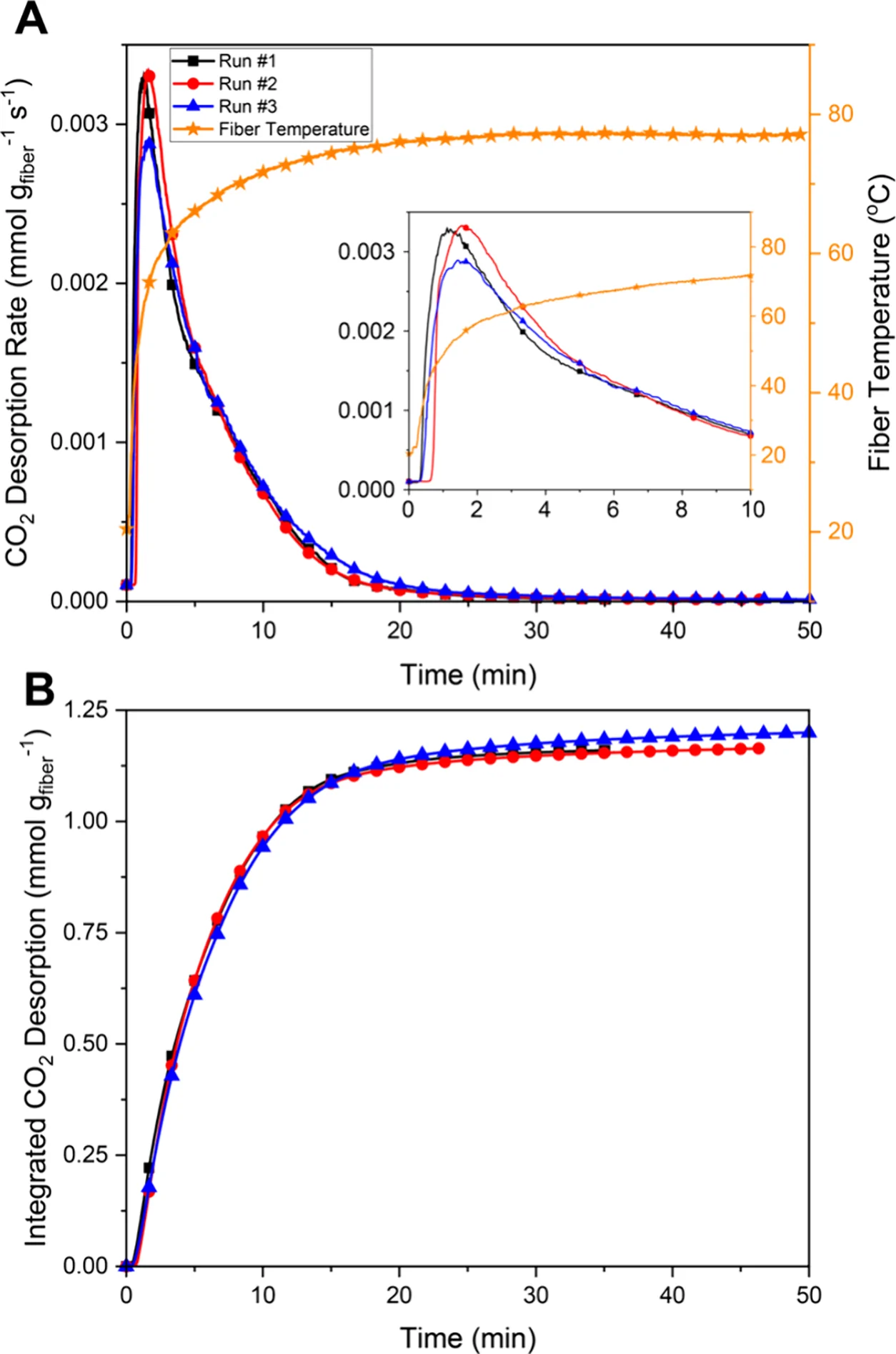
A) CO_2_ desorption rates and B) integrated CO_2_ desorption curves. Sweep gas: N_2_, gas flow rate: 200 sccm, hot water feed temperature: 86 °C.

Based on a target of 90% CO_2_ recovery, a desorption phase time of 13 minutes was estimated from the integrated desorption curves shown in Figure 4. Using the times estimated for the non-adsorptive phases (i.e., desorption, cooling), productivity values may then be calculated using Equation 1.

$$productivity\;\left(mmol\;g^{-1}\;h^{-1}\right)=\frac{{CO}_{2}\;uptake\;\left(mmol\right)}{fiber\;mass\;\left(g\right)\times \left(ads.\;time\;\left(h\right)+non‐ads.\;phase\;time\;\left(h\right)\right)}$$ 1

Figure 5 shows productivity curves generated from the integrated CO_2_ uptakes and a non-adsorptive cycle time estimate of 15 minutes (13 minute desorption + 2 minute cooling) for each breakthrough trial. Estimates revealed an optimum productivity of 1.01 mmol g_fiber_^‑1^ h^‑1^ occurs at an adsorption time of approximately 33 minutes.

**Figure 5:**
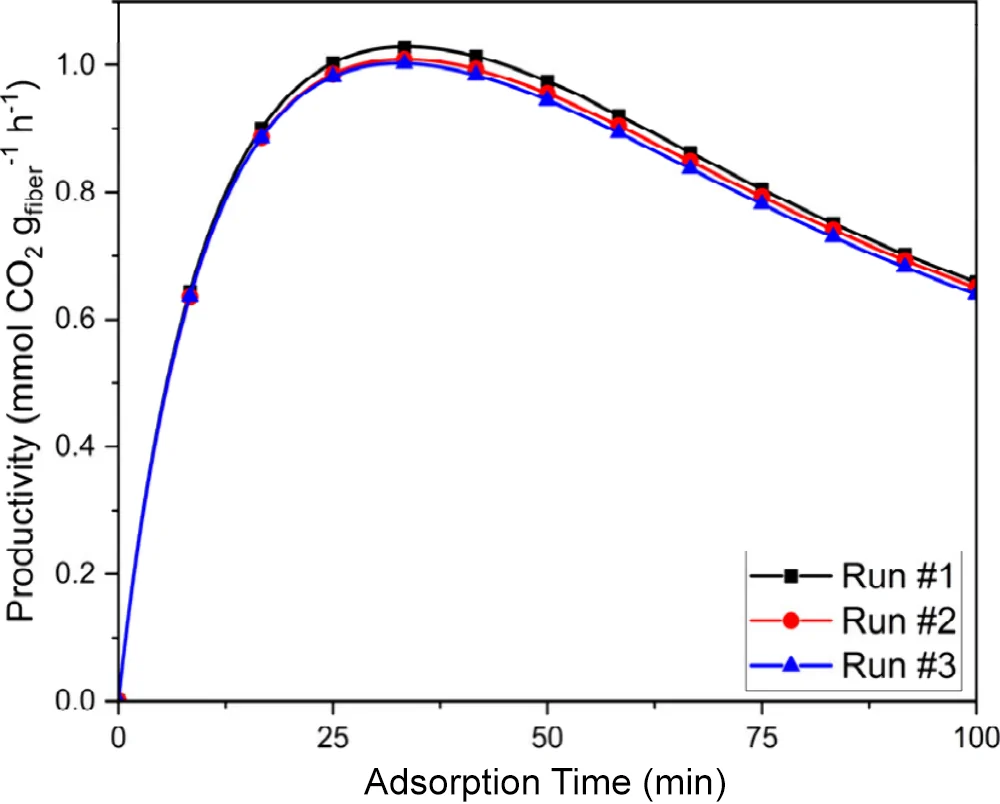
Productivity curves generated using the integrated adsorption data and an estimate of 15 minutes for the non-adsorptive phase times (13 minute desorption + 2 minute cooling).

Using the estimates for the optimum adsorption, desorption, and cooling times, cyclic breakthrough measurements may be completed to mimic a small-scale rapid temperature swing adsorption (RTSA) process. Figure 6 shows the shell-side CO_2_ and H_2_O concentrations for each of the 10 adsorption/desorption cycles under flowing N_2_ sweep. While generally similar in profile, the different cycles exhibited notable variations in the maximum CO_2_ concentration recorded during the different desorption stages, with a sizable decrease observed in the first three cycles, followed by a mostly constant CO_2_ concentration thereafter. This observation is consistent with the adsorption and desorption data presented in greater detail in Figures 7 and 8 and falls in line with prior reports on similar amine infused DAC sorbents**.** More consistent behavior was observed with the shell-side water concentration, with the peak shell-side water concentration during the desorption step remaining generally flat. Small increases were observed in both uptake and peak desorption concentration across the first 5 cycles; however, this is likely attributable to rising ambient temperatures over the first half of the experiment. While the initial spike in shell-side water concentration corresponded to water desorption from the fiber sorbents, a smaller secondary peak was also observed in all cycles, which is likely attributable to increased water vapor diffusion across the lumen barrier with increasing water temperature. Interestingly, the value of the minima between the water peaks and the maximum values of the second peak decreased with subsequent cycles (Figure 6B). This phenomenon may be attributable to cycle-to-cycle changes in the lumen barrier, potentially due to further curing of the styrene-butadiene barrier by diffusing water vapor, or stabilization of the water gradient through the lumen “membrane” over the course of the cycling period.

**Figure 6:**
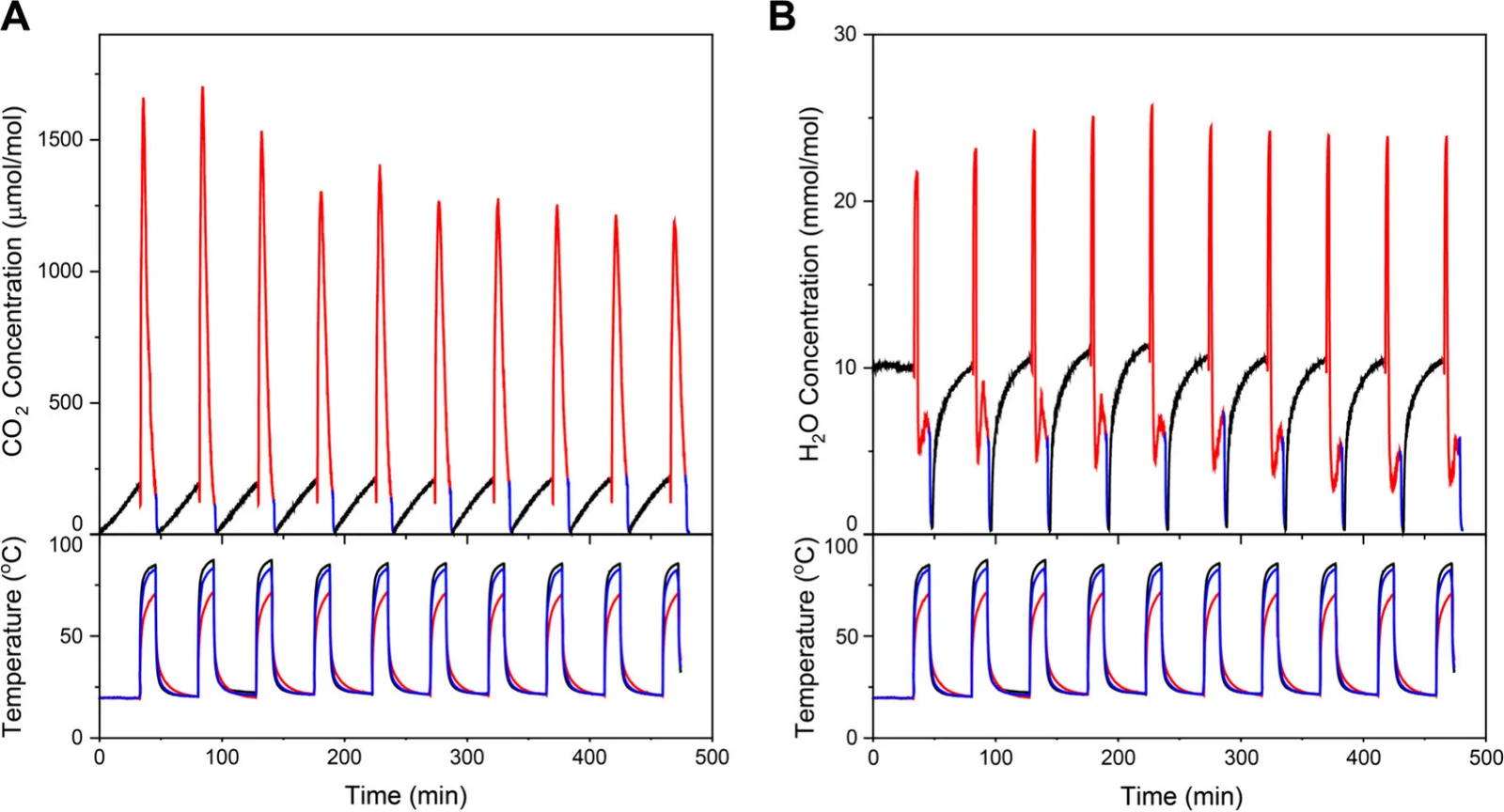
Shell-side concentration measurements for A) CO_2_ and B) water. Black curve: adsorption, red curve: desorption, blue curve: cooling. Lower panel indicates temperatures at the module inlet (black), outlet (blue), and fiber surface (red). Gas flow rate: 1000 sccm, adsorption gas: 400 ppm CO_2_ at 45% RH, desorption gas: dry N_2_, cooling gas: dry N_2_, adsorption temperature: 18-21 °C.

**Figure 7:**
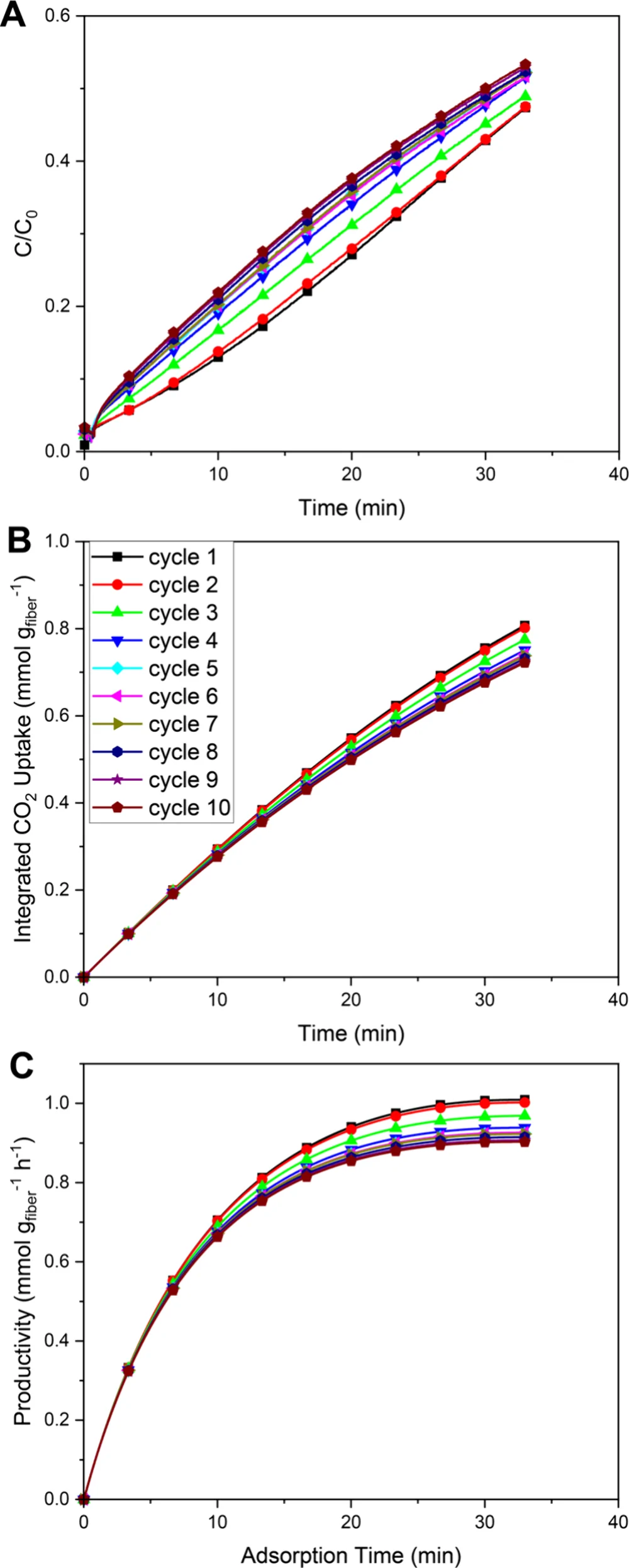
A) Breakthrough curves, B) integrated CO_2_ uptakes, and C) productivity curves for each cycle.

**Figure 8:**
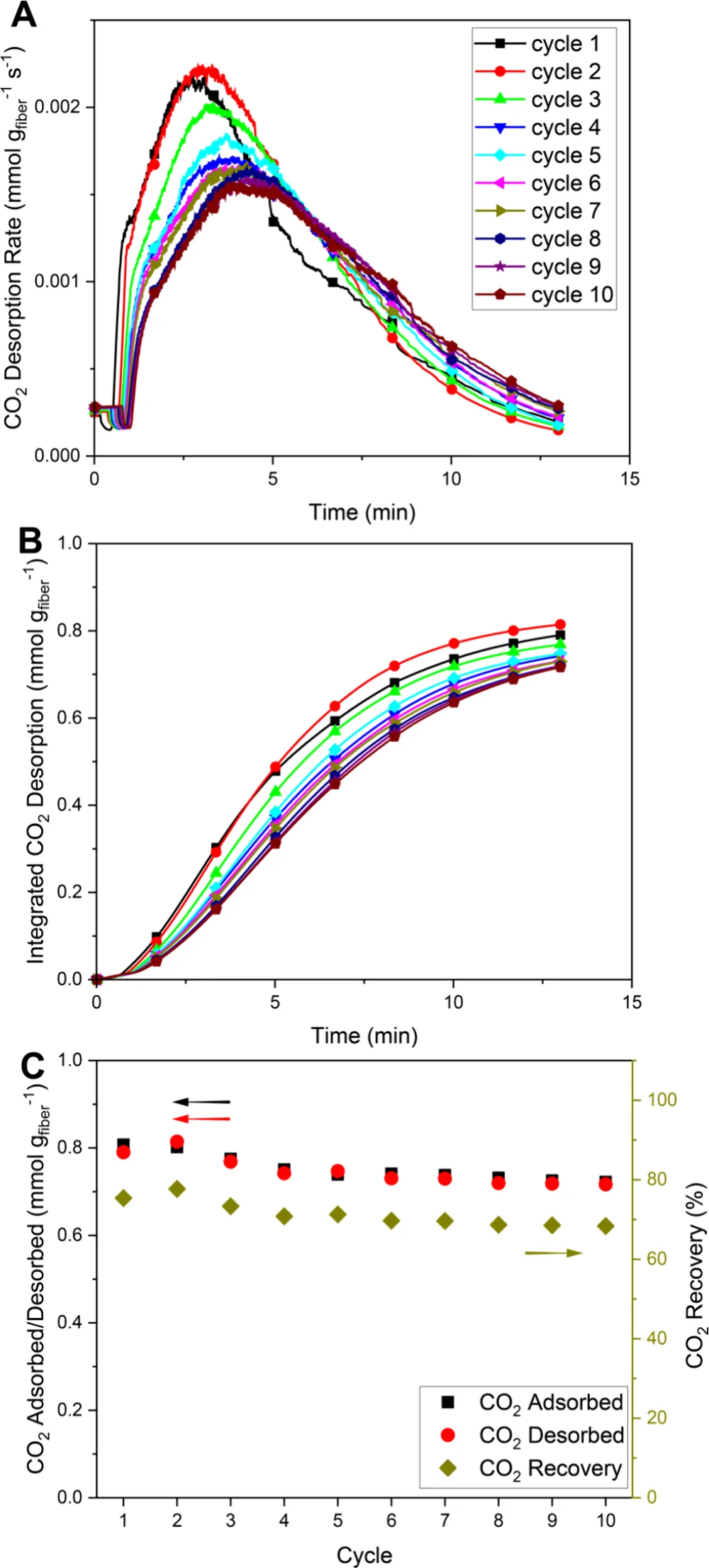
A) CO_2_ desorption rates, B) integrated CO_2_ desorption, and C) adsorption/desorption capacities and CO_2_ recoveries for each cycle.

A compilation of the breakthrough/adsorption data from Figure 6A normalized by feed concentration is presented in Figure 7A. With the abbreviated adsorption period (33 minutes), C/C_0_ only reached ∼0.5 in each cycle, though the final C/C_0_ increased slightly in later cycles. Initial runs (cycles 1-2) exhibited a somewhat sigmoidal shape; however, subsequent cycles became increasingly less sigmoidal, with cycle 3 appearing to be nearly linear and cycles 4-10 becoming more convex. This behavior appears to indicate declining CO_2_ mass transport rates or kinetics, and corresponded to a steady decrease in CO_2_ uptake (Figure 7B), declining from 0.81 mmol g_fiber_^‑1^ in the initial/1^st^ cycle to 0.72 mmol g_fiber_^‑1^ in the 10^th^ cycle. Capacity loss, especially during early cycles, is a common occurrence attributable to PEI repacking and tends to occur in the first few adsorption/desorption cycles,^21^ and is likely responsible for both the decreasing uptakes and worsening kinetics. In this case, the additional water flux from the bore-side water feed may also contribute to accelerated PEI rearrangement, with some potential for increased PEI volatilization or leaching, especially in the event of significant condensation and evaporation during desorption. A similar trend was observed for productivity (Figure 7C) with a drop of approximately 10%, from 1.01 mmol g_fiber_^‑1^ h^‑1^ to 0.90 mmol g_fiber_^‑1^ h^‑1^ between cycles 1 and 10, in line with the cycle-to-cycle capacity loss. For all cycles, the productivity appeared to reach the maximum value, indicating that the selected adsorption, desorption, and cooling times were near optimal for the tested conditions of our fiber formulation.

Figure 8A shows the CO_2_ desorption rate curves for each cycle, which exhibit a similar trend to the adsorption data. The highest desorption rates and narrowest peaks were observed in the first 3 cycles, after which the maximum CO_2_ desorption rate dropped significantly with significant peak broadening suggesting more gradual/staggered CO_2_ desorption. The lower desorption rates were consistent with the decline in CO_2_ uptakes in later cycles, while the widening peaks were likely further evidence of PEI rearrangement negatively impacting CO_2_ liberation into the sweep gas. Integration of these desorption rate curves (Figure 8B) provided the effective swing capacities for each cycle, which ranged from 0.81 to 0.72 mmol g_fiber_^‑1^ and decreased similarly to the uptakes (Figure 8C). These data indicate that the greatest decrease in capacity occurred between cycles 1-4, dropping from 0.81 to 0.75 mmol g_fiber_^‑1^, after which the capacity stabilized around 0.72 mmol g_fiber_^‑1^ in later cycles. CO_2_ recoveries were also calculated from the desorption values and total CO_2_ fed during the adsorption step. The highest recoveries occurred in the first several cycles, peaking at 78% in the second cycle, and then stabilizing at 68% in later cycles. The adsorption/desorption reversibility was also calculated from the uptakes and desorption values, with the lowest reversibility (98%) occurring on the first cycle and an average of >99% (Supporting Information Figure S4).

Water swing capacities were extracted from the water concentration data for comparison with the CO_2_ capacities, which may be found in Figure 9A. The fibers sorbed and desorbed nearly 8 times more water than CO_2_ during each cycle, reaching 6 mmol g_fiber_^‑1^ in later cycles. Interestingly, the water swings increased in later cycles, likely attributable to ambient temperature changes during the course of the testing process, thus causing an increase in absolute humidity in the test modules. Using specific enthalpy of desorption of CO_2_ from PEI (79.5 kJ mol^‑1^)^22^ and the heat of vaporization of water (44.2 kJ mol^‑1^), desorption enthalpies were estimated from the CO_2_ and water swing capacities (Figure 9B). The water desorption required nearly 5 times the energy to desorb than the CO_2_, which is unsurprising considering the disparity between the water and CO_2_ uptakes and swing capacities, as well as the physical properties of water. However, nearly 7 kJ of thermal energy was introduced to the module, significantly greater than the total desorption enthalpy requirement for both the CO_2_ and water desorption. This discrepancy is directly attributable to the substantial disparity between the module casing mass and thermal load relative to that of the fiber sorbents, which can be significantly mitigated in larger scale deployments, where the fiber sorbent mass is significantly increased relative to the mass of the casing.

**Figure 9:**
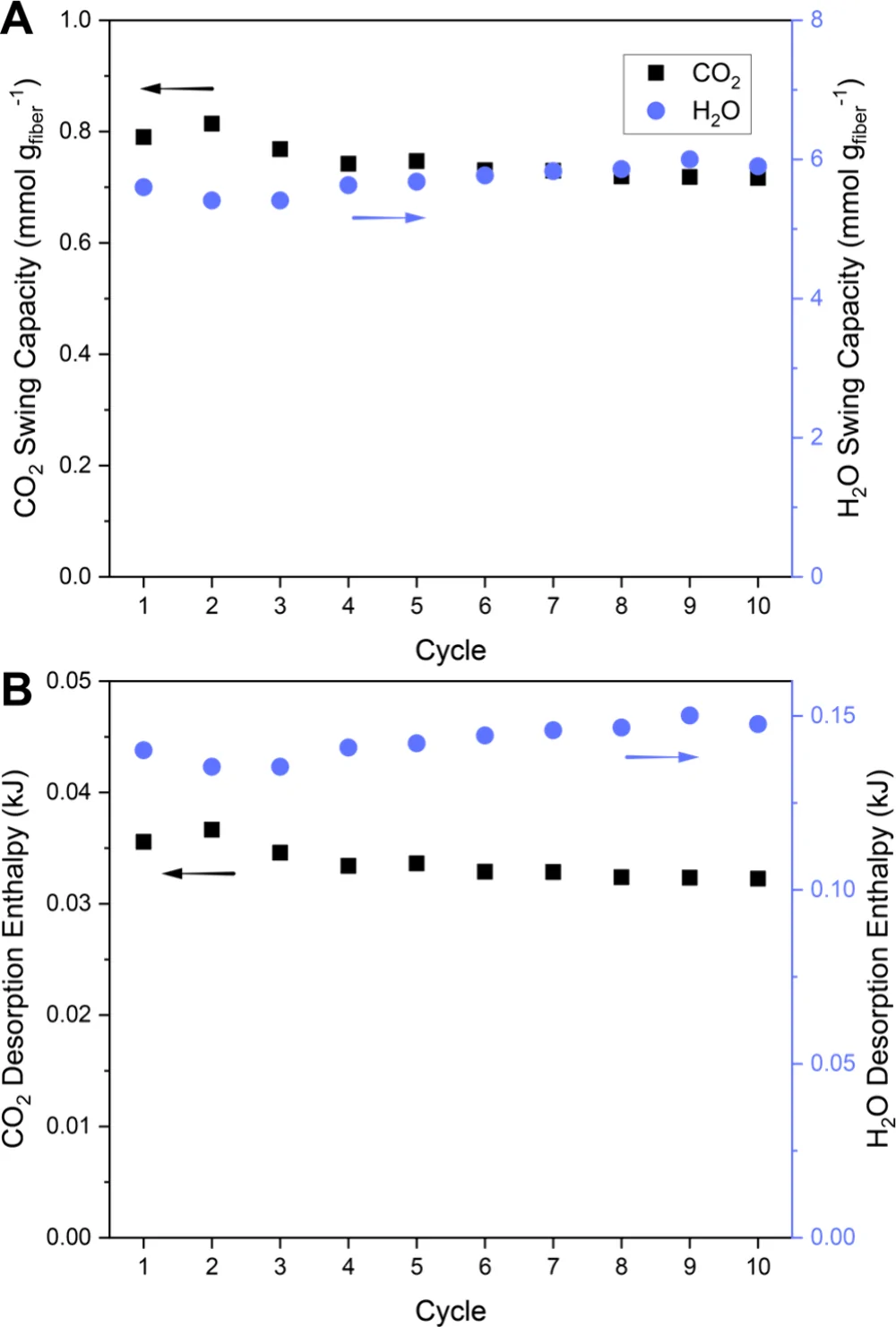
A) Swing capacities and B) desorption enthalpies for H_2_O and CO_2_ by cycle.

Following the cyclic tests, the module was disassembled to retrieve the sorbent fibers for analysis. Approximately half of the fibers were washed with methanol to extract the PEI, and both the PEI-infused and washed fibers were then characterized via N_2_ physisorption and TGA. The PEI and SiO_2_ mass loadings of the fibers, pore volumes, and Brunauer-Emmett-Teller (BET) surface areas of the bare, lumen-coated, and PEI-infused lumen-coated fibers may be found in Table 2. Full N_2_ physisorption isotherms may be found in Supporting Information Figures S5, S6, and S7. The weight fraction of the sorbent (i.e., SiO_2_ + PEI) is relatively low for this formulation of fiber sorbents,^16,17^ though it is vital to note the final sorbent loading is notably impacted both by the presence of the lumen barrier and the reduced PEI fraction compared to previous iterations (0.52 g_PEI_ g_SiO2_^‑1^).

**Table 2:** Fiber Sample Properties

Sample	SiO_2_ wt%	Sorbent wt%	Surface Area (m^2^ g^‑1^)	Pore Volume (cc g^‑1^)
Bare Fibers	47.9 ± 0.1	-	143.1 ± 0.3	0.654
Fibers + Lumen	45.0 ± 0.6	-	126.0 ± 0.4	0.613
Fibers + Lumen + PEI	36.5 ± 0.2	55.5 ± 0.3	57.3 ± 0.1	0.336

## Conclusion

Hollow fiber sorbents were prepared using a dry-jet, wet-quench spinning procedure and their viable deployment in rapid temperature swing adsorption (RTSA) DAC was demonstrated using low thermal intensity desorption with hot water as the thermalizing fluid. A new hollow fiber formulation bearing a hybrid PVDC)/styrene-butadiene lumen barrier was introduced to prevent water flux through the fiber walls, and PEI was infused into the fibers using a continuous-flow infusion procedure. Heating and cooling curves were generated using hot and cold water flow rates ranging from 10 to 30 mL min^‑1^ to evaluate the thermal impedance of the fibers. Full-length breakthrough and hot water desorption measurements were used to determine adsorption and desorption times to achieve optimal productivities during cycling. Cyclic RTSA measurements (10 cycles) were completed to explore cycle-to-cycle stability and determine CO_2_ swing capacities (0.72 mmol g_fiber_^‑1^ after 10 cycles), productivities (0.90 mmol g_fiber_^‑1^ h^‑1^ after 10 cycles), and recoveries (68% after 10 cycles). Interestingly, the approximated adsorption, desorption, and cooling times extracted from the standalone breakthrough and desorption measurements proved to be quite close to the actual optimum operating parameters at the temperature and RH studied, even after the initial capacity fade caused by PEI repacking. With additional uptake and desorption data, real-time parameter selection and feed-forward control should be feasible based on real-time measurement of ambient temperature and RH, as well as known sorbent age and rate of degradation.

Key limitations of this study include the relatively low number of RTSA cycles and the small mass of fiber sorbents relative to the module mass. Larger scales are needed to better assess thermal behavior of such systems, where the sorbent significantly outweighs the module casing. Similarly, longer cycling will give better insight into practical sorbent and fiber lifetimes. Another limitation is the limited range of temperatures and relative humidities tested, as humidity and temperature are known to strongly impact CO_2_ uptake with supported amines.^14,23^ Future experiments will seek to test a wider range of adsorption and desorption conditions, as well as strategies for increasing the CO_2_ swing capacity, productivity, and selectivity to further optimize this fiber system for DAC deployment.

## Supplementary Material



## Notes

Shell-and-tube module images, supplemental lumen barrier images, images of PVDC lumen without styrene-butadiene layer, CO_2_ reversibility data, N_2_ physisorption data (PDF)
