# Supplementary material for: Direct Air CO2 Capture via Hot Water Cycling of Poly(ethyleneimine)/Silica Hollow Fiber Sorbents
Source: Ind Eng Chem Res. 2026 Sep 16;65(38):20710–9. doi: 10.1021/acs.iecr.6c02807 (PMC13632434; doi:10.1021/acs.iecr.6c02807)
Supplement: Supplementary file 1 [file ie6c02807_si_001.pdf]

Supporting Information

Direct Air CO_2 Capture via Hot Water Cycling of Poly(ethyleneimine)/Silica Hollow Fiber Sorbents

Zachary S. Campbell, Steven Schlosser, João Marreiros, William J. Koros, Christopher W. Jones, Matthew Realff, Ryan P. Lively*

School of Chemical and Biomolecular Engineering, Georgia Institute of Technology, 311 Ferst Dr, Atlanta, GA 30332, United States

Email: ryan.lively@chbe.gatech.edu

Table of Contents

I. Module Images……………………………………………………………………….S3

II. Lumen Barrier Images…………………………………………………………..S4

III. Images of PVDC Lumen without Styrene-Butadiene Layer…………………………S5

IV. CO_2 Reversibility Data……………………………………………………………….S6

V. N_2 Physisorption Data…………………………………………………………..S7

I. Module Images

Modules were constructed from Swagelok stainless steel tee-junctions. Three shell-side ports were included to accommodate the gas inlet, gas outlet, and the center thermocouple. The fiber bundle (14 fibers) potted into NPT fittings on either end of the active region of the module. **Figure S1** shows a potted end, the fiber bundle through one of the ports, and the complete module. The fraction of the module was calculated to be 45.5%.

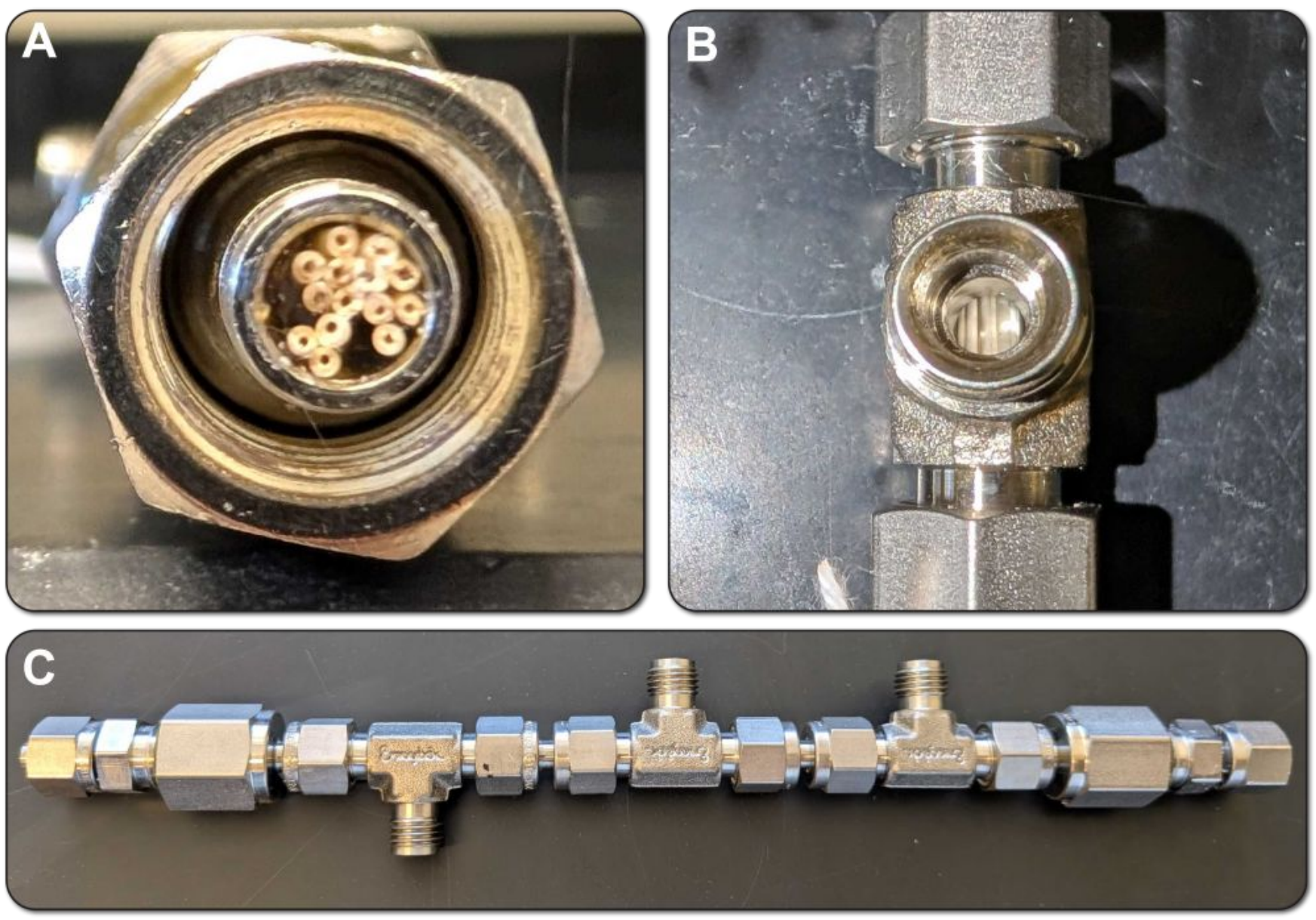

Figure S1. Images of a representative module. A) Module end with potted fibers. B) Fiber bundle inside the module. C) Complete 3-port module.

II. Lumen Barrier Images

In addition to cross-sectional scanning electron microscopy (SEM) images collected with cryo-fractured fibers, fiber segments were also sliced diagonally to expose the lumen barrier surface for imaging. **Figure S2** presents SEM images of the lumen barrier surface after the cyclic measurements.

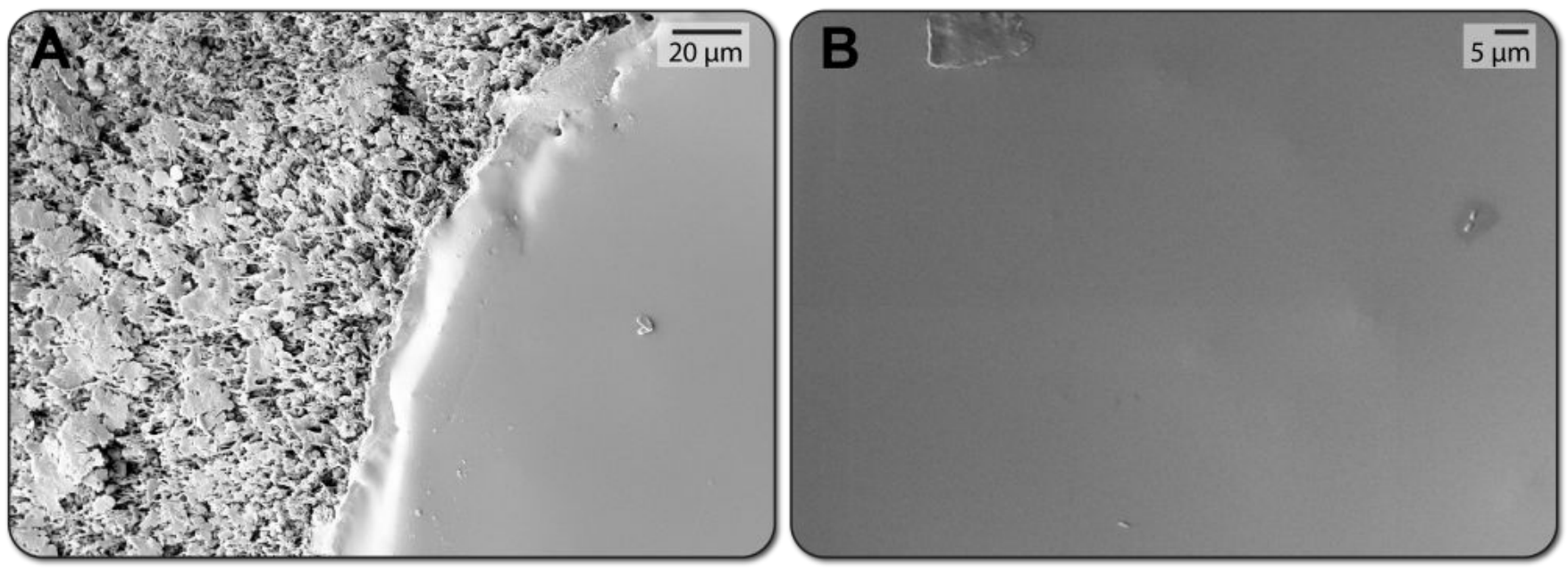

Figure S2. A) Magnified image of lumen-fiber wall interface. B) High magnification SEM image of exposed lumen surface.

III. Images of PVDC Lumen without Styrene-Butadiene Layer

Without the styrene-butadiene secondary coat, the PVDC barrier experienced severe pitting and degradation after exposure to repeated hot water cycling. **Figure S3** shows the PVDC lumen after repeated thermal cycling with hot water.

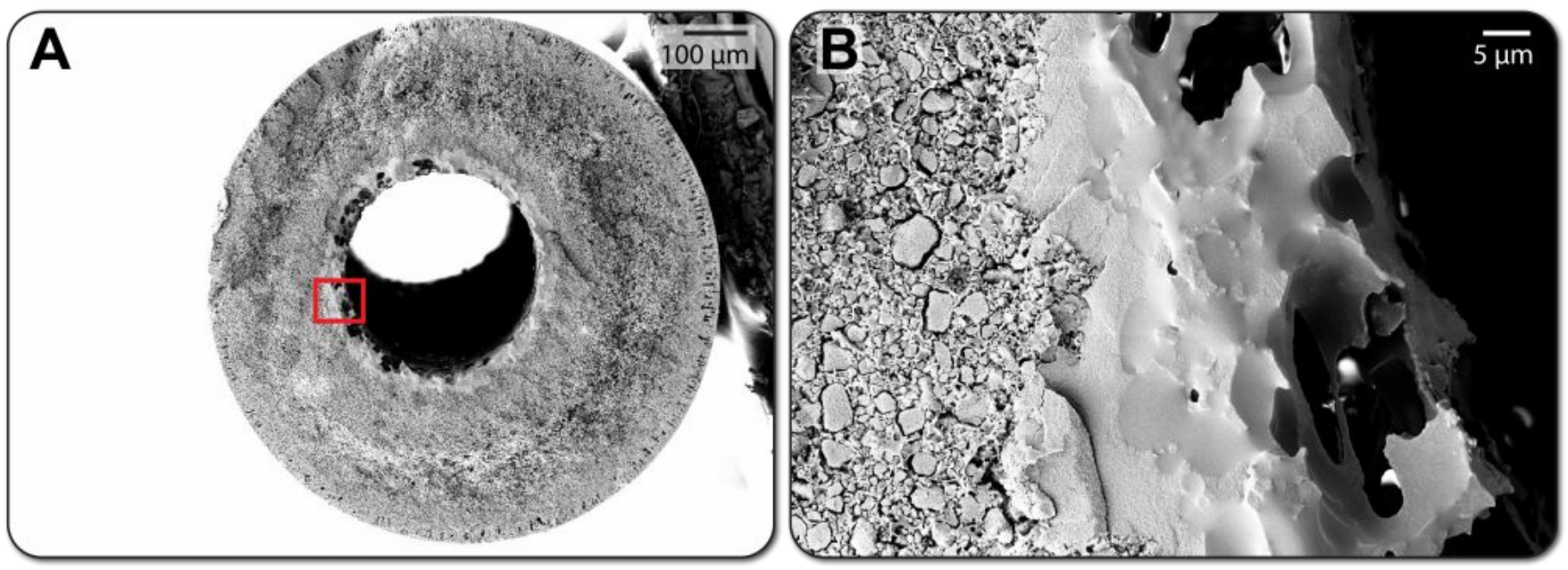

Figure S3. A) Cross section of PVDC-coated fiber after multiple hot water cycles. B) Higher magnification SEM image of the lumen barrier in panel A.

IV. CO_2 Reversibility Data

As all phases of the temperature swing cycle are abbreviated, it is reasonable to expect that a small fraction of the CO_2 may not be removed from the sorbents during the desorption phase. For this reason, the reversibility of the sorbents was calculated by dividing the quantity of CO_2 desorbed by the quantity adsorbed during each cycle (**Figure S4**). Nearly 100% of the CO_2 adsorbed by the fiber sorbents was recovered during the desorption step in all cycles.

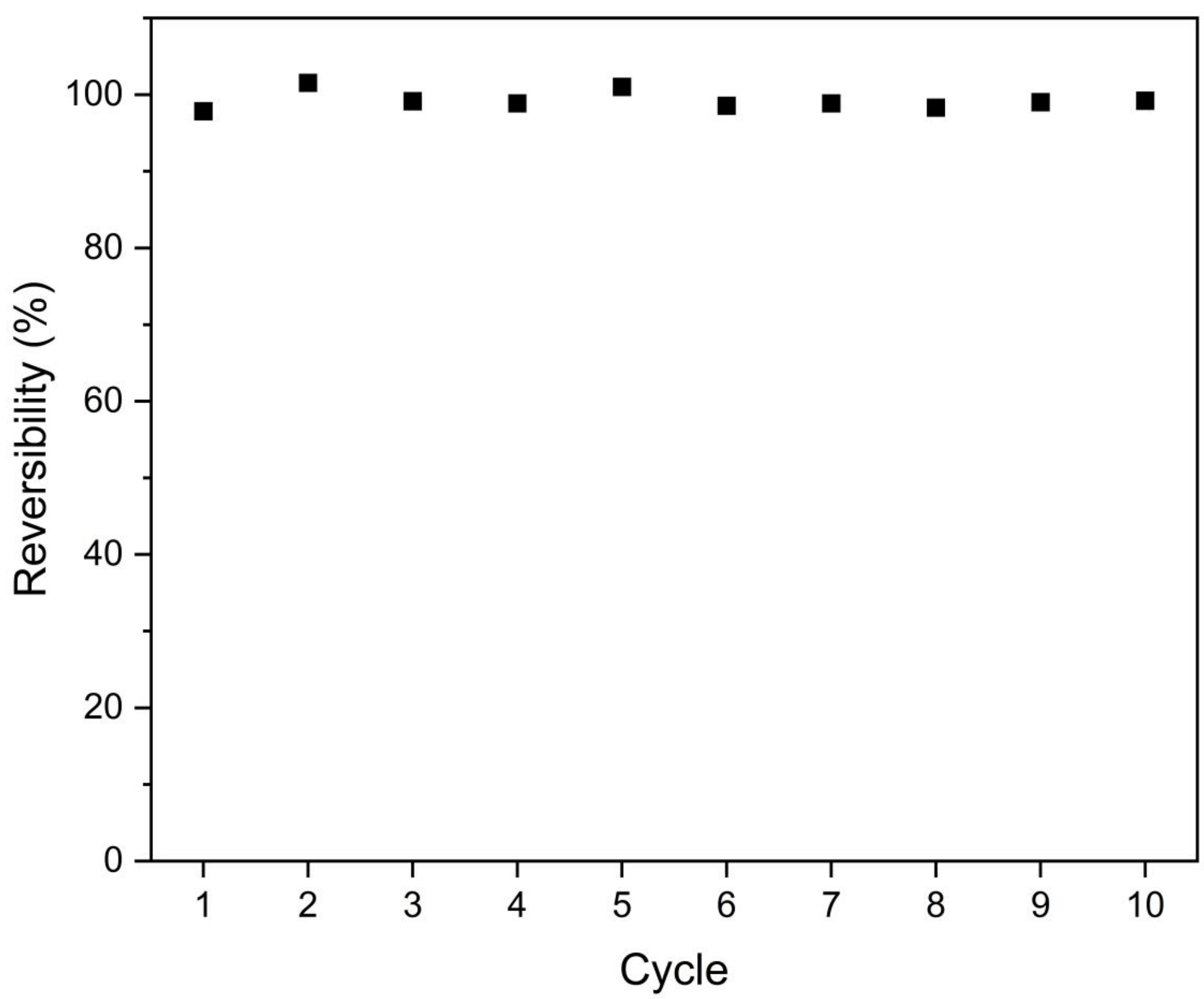

Figure S4. Plot of the cycle-to-cycle reversibility calculated from the quantities of CO_2 adsorbed and desorbed.

V. N_2 Physisorption Data

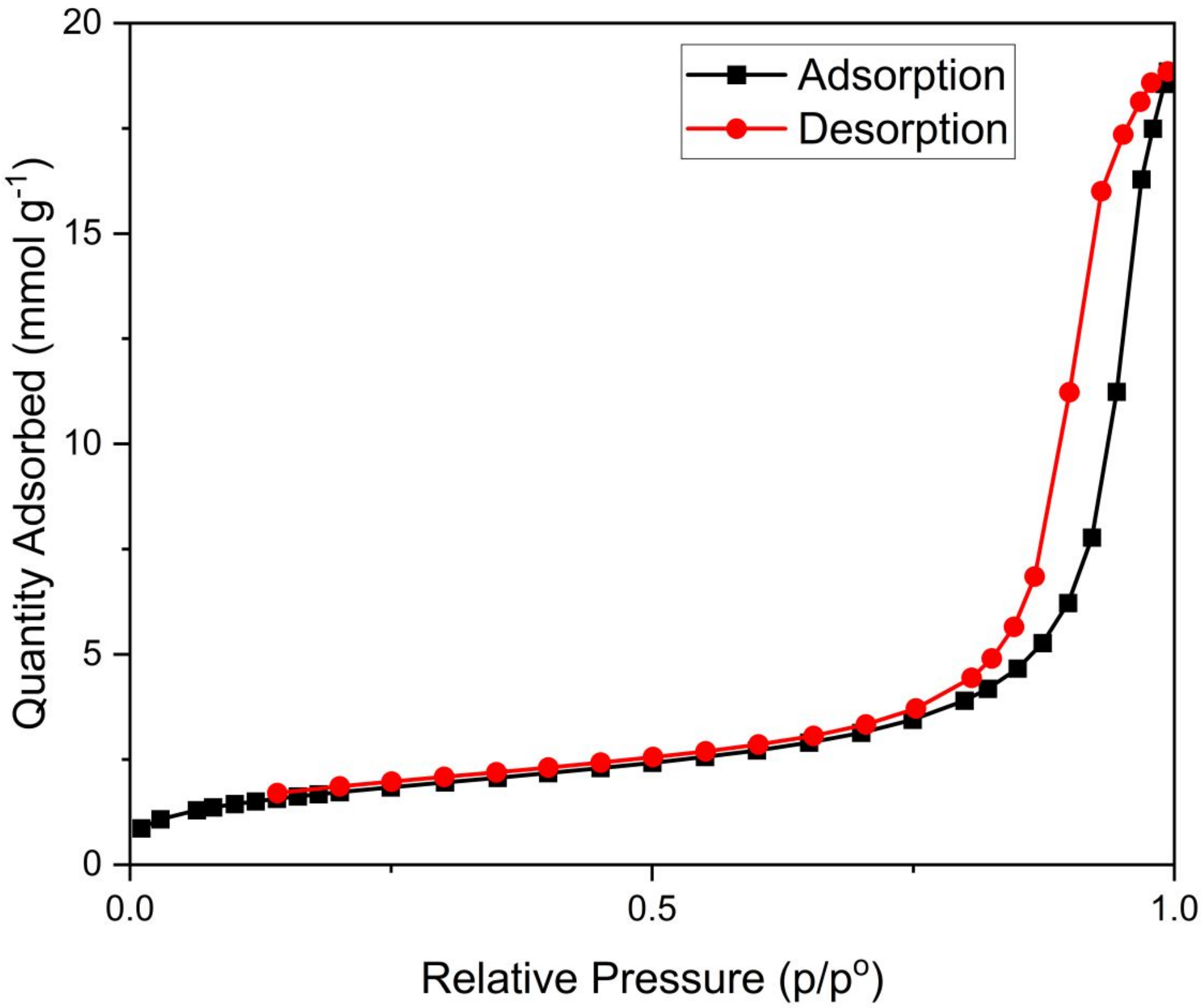

Figure S5. N_2 isotherm of the bare SiO_2-PSF fibers.

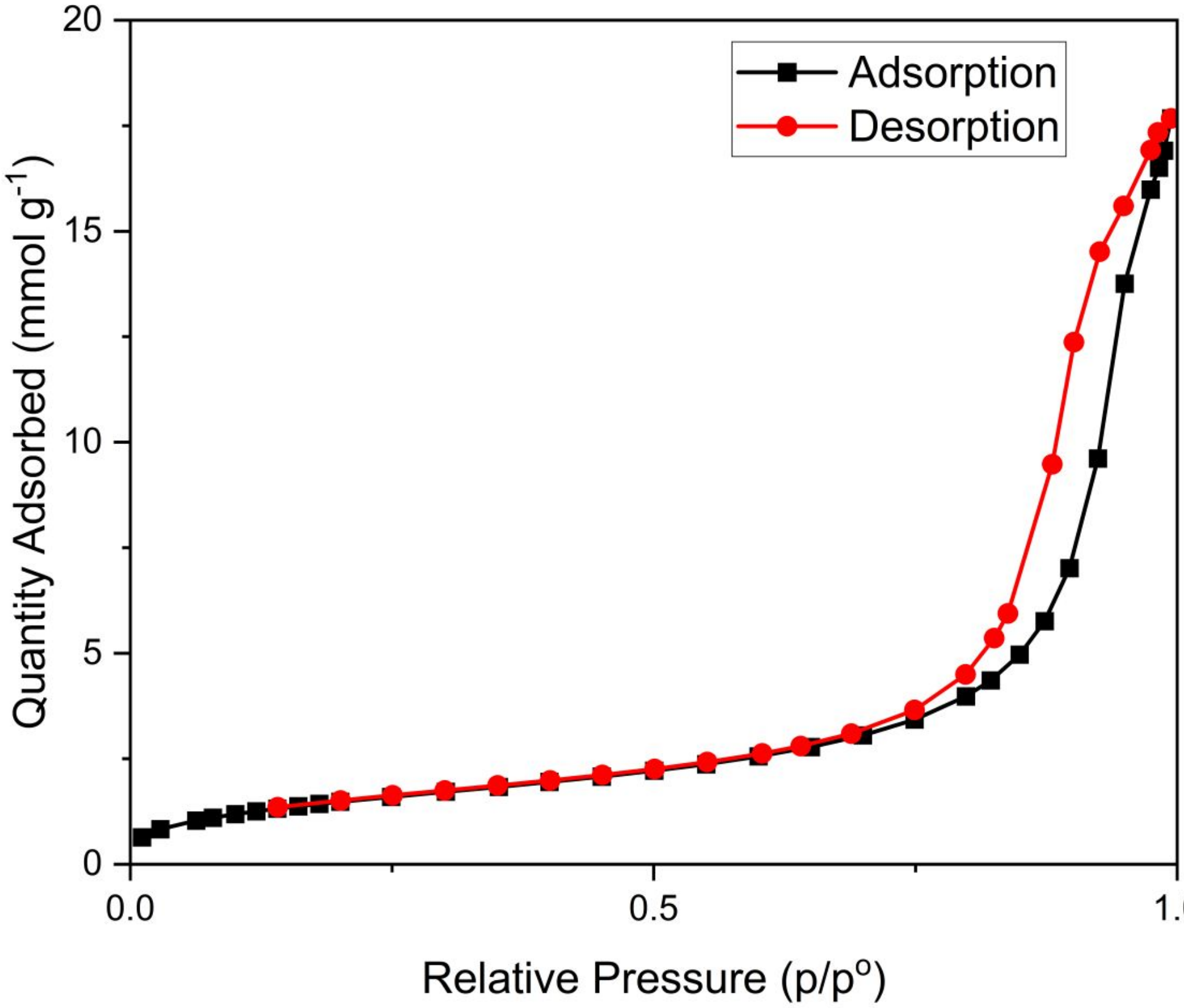

Figure S6. N_2 isotherm of the SiO_2-PSF fibers with the hybrid PVDC/styrene-butadiene lumen barrier installed.

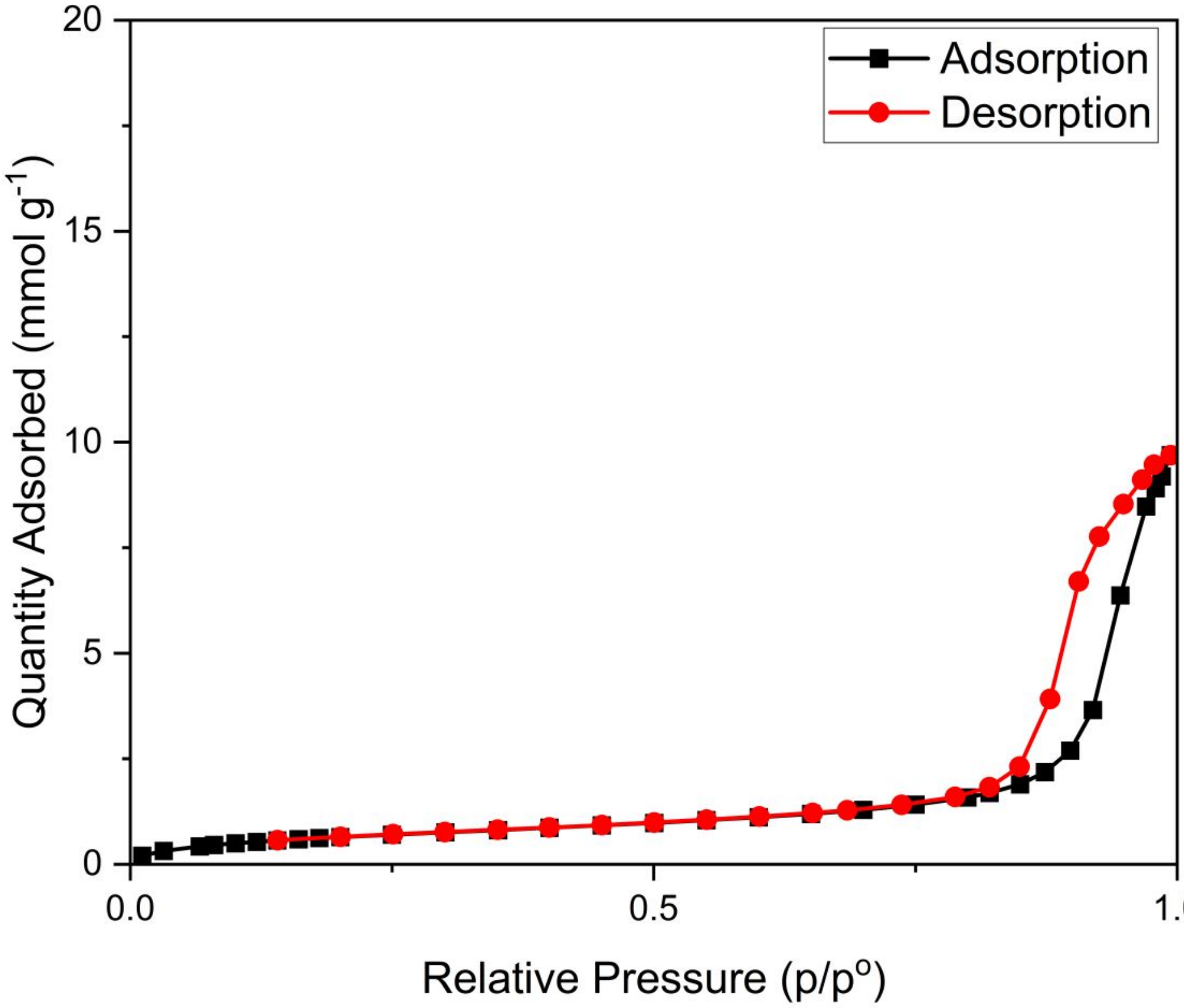

Figure S7. N_2 isotherm of the PEI-infused fibers with the PVDC/styrene-butadiene barrier.
